# Multiplatform Genomic and Transcriptomic Profiling of Breast Carcinomas with an Invasive Micropapillary Component

**DOI:** 10.3390/genes17101190

**Published:** 2026-09-25

**Authors:** Boram Lee, Je-Gun Joung, Woong-Yang Park, Hyunwoo Lee, Eun Yoon Cho

**Affiliations:** 1 Department of Pathology and Translational Genomics, Samsung Medical Center, Sungkyunkwan University School of Medicine, Seoul 06351, Republic of Korea; 2 Department of Biomedical Science, College of Life Science, CHA University, Seongnam 13488, Republic of Korea; 3 Department of Health Sciences and Technology, Samsung Advanced Institute for Health Sciences & Technology, Sungkyunkwan University, Seoul 06351, Republic of Korea

**Keywords:** invasive micropapillary carcinoma, breast cancer, micropapillary morphology, *ERBB2*, HER2, copy-number alteration, targeted sequencing, whole-exome sequencing, RNA sequencing

## Abstract

**Background/Objectives:** Invasive micropapillary carcinoma (IMPC) of the breast is a rare subtype with a strong propensity for lymph node metastasis, but its molecular features remain incompletely defined. We characterized the genomic and transcriptomic features of breast carcinomas with an invasive micropapillary component. **Methods:** Thirteen tumors with a micropapillary component of at least 10%, including four pure IMPCs, were analyzed by targeted sequencing (*n* = 11), whole-exome sequencing with array comparative genomic hybridization (*n* = 2), and whole-transcriptome sequencing (*n* = 9). Alteration frequencies were compared with TCGA-BRCA across all 375 panel genes, with multiple-testing correction and a sensitivity analysis restricted to truncating and hotspot variants. **Results:** *TP53* was the most frequently altered gene (72.7%), followed by *ERBB2* amplification (45.5%); *PIK3CA* and *GATA3* were each altered in 27.3%. Four rarely mutated genes were more frequent than in TCGA-BRCA after correction, but these differences were based on few tumors and predominantly on uncertain missense variants and were not retained under the stringent definition. Recurrent high-level amplification was concentrated in 17q12-q21, and both pure IMPCs analyzed by array comparative genomic hybridization showed 8q gain. All five tumors with panel *ERBB2* amplification were HER2-positive by immunohistochemistry, and TCGA-anchored PAM50 classified all three HER2-positive transcriptomes as HER2-enriched. Fourteen fusion transcripts were detected, none recurrent. **Conclusions:** These findings support a heterogeneous molecular landscape characterized by established breast cancer drivers, recurrent copy-number changes involving 8q and 17q, predominantly private fusion events, and cross-platform concordance of *ERBB2* amplification with HER2 protein expression and PAM50 HER2-enriched classification.

## 1. Introduction

Invasive micropapillary carcinoma (IMPC) is a rare subtype of breast cancer characterized by small clusters of tumor cells surrounded by clear stromal spaces and an inside-out growth pattern, in which the apical surface of the neoplastic cells faces the stroma. A micropapillary component is present in approximately 2–8% of invasive breast carcinomas, whereas pure IMPC is considerably less common [1]. IMPC has a strong propensity for lymphovascular invasion and lymph-node metastasis, even when the micropapillary component is limited [2,3].

Most IMPCs are hormone receptor positive, whereas reported HER2 positivity varies across series, in part because the inside-out architecture produces an incomplete, basolateral membrane staining pattern that complicates scoring [4]. Genomic studies have repeatedly identified broad copy-number alterations, including gains of 8q and 17q, whereas the recurrent driver mutations largely overlap those of conventional breast carcinoma [5,6]. More recent genomic, spatial transcriptomic, and single-cell studies have revealed substantial molecular heterogeneity in IMPC, with recurrent copy-number alterations and evidence of clonal evolution during lymph-node dissemination [7,8].

Although molecular studies of IMPC remain limited by small cohort sizes, they now span targeted sequencing, whole-exome and whole-genome sequencing, bulk and spatial transcriptomics, and single-cell approaches [5,6,7,8,9]. No defining recurrent molecular alteration has emerged, and reported mutation and copy-number frequencies vary considerably across series. Here, we present an exploratory, integrative molecular characterization of a single-institution series of breast carcinomas with an invasive micropapillary component, using multiple genomic and transcriptomic platforms with case-level cross-platform integration where available. We interpret the findings in the context of previous molecular studies of IMPC and use TCGA-BRCA as an external reference for exploratory comparison.

## 2. Materials and Methods

### 2.1. Patients and Samples

Sixteen breast carcinomas with a documented micropapillary component were selected based on the availability of non-formalin-fixed tissue for molecular profiling. Two tumors resected in 2008, both pure IMPCs, were analyzed by whole-exome sequencing, array comparative genomic hybridization, and whole-transcriptome sequencing; matched normal breast tissue was available for both. Seven tumors resected between 2010 and 2014 were those for which fresh–frozen tissue was available from the institutional biobank and underwent targeted panel sequencing; six of them also underwent whole-transcriptome sequencing. Seven tumors sampled between 2015 and 2016 underwent targeted panel sequencing, and two of them also underwent whole-transcriptome sequencing. All targeted panel and whole-transcriptome sequencing was performed during 2015–2016. Where RNA was available, it was extracted from the same specimen used for DNA extraction.

The study cohort was restricted to tumors in which micropapillary morphology accounted for at least 10% of the invasive carcinoma, the threshold used at our institution to report a mixed carcinoma with a micropapillary component. Three tumors with a recorded micropapillary component of 5% were excluded, leaving 13 tumors: four pure IMPCs (100% micropapillary component) and nine mixed tumors. The proportion of micropapillary morphology, tumor purity, tumor size, nodal status, and lymphovascular invasion were assessed on hematoxylin and eosin-stained sections by the study pathologist (E.Y.C.). Tumor cellularity was estimated histologically with reference to the purity estimate generated by the sequencing pipeline. HER2 status was determined by review of the original HER2 immunohistochemistry and in situ hybridization results by the study pathologist (E.Y.C.) according to the 2018 ASCO/CAP HER2 guideline [10]. Estrogen and progesterone receptor status was assessed according to the ASCO/CAP hormone receptor guideline [11], and hormone receptor positivity was defined as positivity for either receptor. Ki-67 was recorded as a four-grade labeling index (1+, 0–25%; 2+, 26–50%; 3+, 51–75%; 4+, 76–100% of tumor nuclei). The study was approved by the Institutional Review Board of Samsung Medical Center (SMC 2012-07-036-005; date of approval: 7 August 2012).

### 2.2. Nucleic Acid Extraction

Genomic DNA was extracted from fresh tissue or fresh–frozen tissue with the QIAamp DNA Mini Kit (Qiagen, Hilden, Germany). DNA quantity and quality were assessed with a NanoDrop 8000 spectrophotometer (Thermo Fisher Scientific, Wilmington, DE, USA), a Qubit 2.0 fluorometer (Invitrogen, Carlsbad, CA, USA), and a 2200 TapeStation (Agilent Technologies, Santa Clara, CA, USA). Total RNA was extracted with the RNeasy Mini Kit (Qiagen).

### 2.3. Targeted Deep Sequencing and Variant Analysis

Eleven tumors were profiled with CancerSCAN version 2, the 375-gene targeted sequencing panel used for clinical breast cancer testing at our institution, as previously described [12]. Captured libraries were sequenced as 100 bp paired-end reads on a HiSeq 2500 (Illumina, San Diego, CA, USA). Per-sample mean target coverage, on-target rate, and duplication rate are given in Supplementary Table S2. Reads were aligned to hg19 with BWA-MEM v0.7.5; single-nucleotide variants were called as the union of MuTect v1.1.4 and LoFreq v0.6.1 calls, and small indels were called with Pindel v0.2.4, and gene-level copy number was estimated from exon read depth normalized to the target-region coverage of each sample. The pipeline reports variants with a variant allele fraction of at least 0.02 and at least four supporting reads, excludes population polymorphisms with a minor allele frequency of 0.001 or more in its population allele-frequency annotation [12] and, in this study, in gnomAD v2.1.1, while retaining recurrent somatic hotspots, and applies an allele-frequency cutoff of 0.03 in an internal panel of 400 ethnicity-matched normal samples and an indel length limit of 30 bp. Amplification was defined as an estimated copy number of at least 6 and deletion as a copy number below 0.7. *APOBEC3B* deletion calls were excluded because the common germline deletion polymorphism at this locus, which is frequent in East Asian populations [13], cannot be distinguished from somatic loss without matched normal DNA. Structural variants were credited to both annotated partner genes.

Because matched normal DNA was not available for the panel-profiled tumors, retained variants were analyzed as putative somatic variants at the cohort level, although germline origin could not be excluded for individual variants. All retained variants were reannotated using MyVariant.info (hg19; queried 17 September 2026 against the index built 24 June 2025) for gnomAD v2.1.1 exome and genome allele frequencies, ClinVar (May 2025 release), and COSMIC (v68 as distributed with the index). Variants were also matched to the cancer hotspot catalog of Chang et al. (cancerhotspots.org, version 2, downloaded 17 September 2026) [14]. Population database status, ClinVar category, and variant classification are provided in Supplementary Table S3. For sensitivity analysis, a stringent variant set was defined as truncating variants (frameshift, nonsense, or splice-site) or variants at a cancerhotspots residue, excluding variants observed in population databases based on the original pipeline annotation or gnomAD v2.1.1 exome/genome data unless they represented recurrent somatic hotspots. Recurrent somatic hotspots were defined as variants at a cancerhotspots residue, or variants reported at least five times in breast cancer in COSMIC.

### 2.4. Whole-Exome Sequencing and Array Comparative Genomic Hybridization

Two pure IMPC tumors with matched normal tissue underwent whole-exome sequencing with SureSelect XT Human All Exon v5 libraries (Agilent Technologies) on a HiSeq 2500. Reads were aligned to hg19 with BWA v0.6.2, and processed with Picard v1.8 and GATK v2.2.9. Variants were called with GATK and MuTect v1.1.4, and annotated with ANNOVAR. Somatic calls with a gnomAD v2.1.1 allele frequency of 0.001 or more were excluded. Mean target coverage and the fraction of target bases covered at 20-fold, from Picard CalculateHsMetrics, are given in Supplementary Table S2. The same tumors were analyzed by array comparative genomic hybridization on the SurePrint G3 Human CGH Microarray, 1 × 1M (Agilent Technologies), with segmentation in GLAD.

### 2.5. Whole-Transcriptome Sequencing and Expression Analysis

Whole-transcriptome sequencing was performed on nine tumors. Libraries were prepared from two micrograms of total RNA with the TruSeq RNA Sample Preparation v2 kit (Illumina) and sequenced as 100 bp paired-end reads on a HiSeq 2500 at two sequencing sites: six libraries by an external sequencing provider and three at the Samsung Genome Institute. Reads were aligned to hg19 with TopHat v2.0.6, gene-level counts were generated with HTSeq v0.6.1, and fusion transcripts were called with deFuse, excluding mitochondrial calls and calls involving unannotated clone contigs. Library-level quality metrics are given in Supplementary Table S2. Gene-level RNA-seq read counts for the nine tumors and two matched normal samples are provided in Supplementary Data S1.

Genes with at least 10 counts in at least three libraries were retained for analysis (16,558 genes). Raw counts were analyzed with DESeq2 for differential expression [15], and variance-stabilized expression values were used for expression summaries, intrinsic subtyping, correlation analysis, and principal-component analysis. For the HER2-enriched contrast, sequencing site was included as a covariate using the design formula ~ site + subtype, with site coded as external provider versus Samsung Genome Institute. As sensitivity analyses, the contrast was repeated without a site term and after restricting the analysis to the six libraries sequenced by the external provider. Technical structure was assessed by principal-component analysis of the 2000 most variable genes, and each principal component was tested for association with sequencing site, HER2 immunohistochemistry, and micropapillary percentage.

Intrinsic subtype was assigned using the PAM50 centroid classifier [16] with TCGA-BRCA as the external reference. The reference comprised RNA-seq data from 1102 TCGA-BRCA primary tumors (GDC HTSeq gene counts, downloaded July 2016 [17]), which were variance-stabilized with DESeq2 and mapped to HGNC gene symbols. Each tumor in the present series was quantile-normalized across the 14,014 genes shared with the reference to the mean quantile distribution derived from TCGA-BRCA, and each PAM50 gene was centered on the TCGA-BRCA median. Subtype was assigned according to the highest Spearman correlation with the published PAM50 centroids. Forty-nine of the 50 PAM50 genes were available; *MIA* did not pass the expression filter, and *NUF2*, *NDC80*, and *ORC6* were matched to the centroid symbols *CDCA1*, *KNTC2*, and *ORC6L*, respectively. The correlation with the assigned centroid and the difference between the highest and second-highest centroid correlations were reported as measures of classification confidence.

The single-sample AIMS classifier [18], implemented in genefu [19], was also applied. AIMS uses within-sample gene-pair rules and does not require cohort-level centering. The classifier was applied to 2^VST^ values. Associations between gene expression and the proportion of micropapillary morphology were assessed gene by gene using the Spearman rank correlation across the nine tumors.

### 2.6. Comparison with a Public Reference Cohort

Alteration frequencies were compared with the TCGA breast invasive carcinoma cohort (PanCancer Atlas; 1066 samples with mutation data and 1070 with copy-number data) [17], retrieved through cBioPortal [20,21]. The gene universe was predefined as all 375 genes targeted by the panel, each of which was represented in the TCGA-BRCA exome mutation data available through cBioPortal, and multiple-testing correction was applied across all 375 genes. Only protein-altering variants were counted in both cohorts. The comparison used the panel variants retained by the original filtering pipeline and all protein-altering somatic variants in TCGA-BRCA. A sensitivity analysis was then performed using the stringent variant set defined above, with the same truncating-or-hotspot rule applied to TCGA-BRCA. High-level amplifications and deep deletions were compared with TCGA-BRCA GISTIC calls, with multiple-testing correction across the same 375-gene universe.

Because mutation frequencies vary across breast cancer subtypes, the mutation analysis was also repeated within surrogate receptor-defined subtypes, with correction across all gene-by-stratum tests. Tumors were classified as hormone receptor-positive/HER2-negative, HER2-positive, or triple-negative according to receptor status, and the same definitions were applied to the TCGA cohort using the breast cancer study reporting immunohistochemical receptor status, in which 859 sequenced tumors could be assigned to these groups [22]. All five HER2-positive tumors were analyzed as a single HER2-positive stratum regardless of hormone receptor status.

### 2.7. Statistical Analysis

Alteration frequencies were compared by Fisher’s exact test, with the conditional maximum likelihood odds ratio and its 95% confidence interval, and continuous variables by the Wilcoxon rank-sum test. Continuous data are reported as median and range. *P*-values were adjusted by the Benjamini–Hochberg method within each analysis, over the predefined gene universe where applicable, and both nominal *p*-values and false discovery rates are reported. Analyses used R version 4.3.2.

## 3. Results

### 3.1. Patients and Samples

Thirteen tumors were included (Table 1). All patients were women, with a median age at diagnosis of 49 years (range 37–80). The median micropapillary component was 40% (range 10–100%), and four tumors (30.8%) were pure IMPC. Median tumor size was 3.8 cm (range 1.2–14.0 cm, *n* = 11). Ten tumors (76.9%) were node-positive, with a median of 8 positive nodes (range 0–26), and 8 (61.5%) had four or more positive nodes. Lymphovascular invasion was present in all 11 evaluable tumors (unknown in 2), hormone receptor positivity in 10 (76.9%), and HER2 positivity in 6 (46.2%). Targeted deep sequencing data were available for 11 tumors, whole-exome sequencing and array comparative genomic hybridization for two, and whole-transcriptome sequencing for nine; case-level platform coverage is given in Supplementary Table S1, and per-sample sequencing quality is given in Supplementary Table S2.

### 3.2. Targeted Deep Sequencing

Eleven tumors were profiled on the 375-gene panel. The filtered variant list contained 87 small variants (70 missense substitutions, three nonsense substitutions, four splice-site variants, and 10 indels, nine of them frameshift), 26 high-level amplifications, nine deletions, and six structural variants. The median number of small variants per tumor was 7 (range 4–17) and the median number of gene-level copy-number changes was 1 (range 0–9). Case-level variants are listed with their population database status, hotspot, and ClinVar annotation in Supplementary Table S3: 39 of the 87 small variants have an entry in a population database, and 22 met the stringent definition.

The most frequently altered gene was *TP53*, altered in 8 of 11 tumors (72.7%; seven small variants and one deletion), followed by *ERBB2* amplification in 5 of 11 (45.5%) and *BRCA2* in 4 of 11 (36.4%); *DOCK2*, *GATA3*, *NOTCH1* and *PIK3CA* were each altered in 3 of 11 (27.3%) (Figure 1). *BRCA2*, *DOCK2*, *NOTCH1,* and *PIK3CA* were altered by small variants and *ERBB2* only by amplification; *GATA3* carried two small variants and one tandem duplication. Two of the three *PIK3CA* variants were hotspot substitutions, whereas the third was a non-hotspot missense variant.

Recurrent high-level amplification was confined to the 17q12-q21 region, which contains *ERBB2* and *CDK12* at 17q12 and *RARA* at 17q21.2: *ERBB2* was amplified in 5 of 11 tumors and *CDK12* and *RARA* in 2 each, and 7 of 11 tumors carried at least one amplification. The nine deletions were single events involving *TP53*, *CDH1*, *CTCF*, *CHEK2*, *DNMT1*, *EP300*, *KEAP1*, *RAD51C* and *RPA1*.

Six structural variants were detected in 5 of 11 tumors, none recurrent: tandem duplications involving *BCR*, *GATA3* and *MLH1*, deletions involving *ETV6* and *PTEN*, and an inversion joining *TEX2* and *GNA13* (Supplementary Table S4). The *PTEN* deletion and the *GATA3* tandem duplication affect established breast cancer genes.

### 3.3. Comparison with a Public Reference Cohort

Mutation frequencies were compared with TCGA-BRCA across all 375 panel genes, with multiple-testing correction applied across the full gene set (Figure 2; Supplementary Table S5). Frequencies of canonical breast cancer genes were generally similar to those in the reference cohort: *PIK3CA* was mutated in 3 of 11 tumors (27.3%) against 32.6% of TCGA-BRCA (*p* = 1.00), *GATA3* in 2 of 11 (18.2%) against 11.9% (*p* = 0.63), and *CDH1*, *MAP3K1* and *PTEN* were unmutated against 12.2%, 8.4% and 5.4% (*p* = 0.38, 0.61 and 1.00). *TP53* was more frequent in this cohort (7 of 11, 63.6% versus 32.6%), but the difference was only nominally significant (*p* = 0.047) and did not remain significant after multiple-testing correction (FDR = 0.83).

Four genes had FDR < 0.05 in the broader comparison: *NOTCH1* (3 of 11, 27.3% vs. 0.75% in TCGA-BRCA), *BRCA2* (4 of 11, 36.4% vs. 2.7%), *FLT4* (2 of 11, 18.2% vs. 0.09%), and *DOCK2* (3 of 11, 27.3% vs. 1.2%) (Figure 2; Supplementary Table S5). Because the reference frequencies were below 3%, these differences were based on only two to four tumors. The 12 variants underlying these comparisons comprised 10 missense substitutions and two frameshift variants, and seven were recorded in population databases (Supplementary Table S3). Under the stringent definition restricted to truncating and hotspot variants in both cohorts, no gene remained significant after multiple-testing correction (minimum FDR = 1.0).

*ERBB2* amplification was observed in 5 of 11 tumors (45.5%) versus 11.5% of TCGA-BRCA (*p* = 0.006), but the difference was not significant after multiple-testing correction (FDR = 1.0). *CDK12*, in the same amplicon, was amplified in 2 of 11 (18.2%) versus 10.4% (*p* = 0.32). The *ERBB2* amplification frequency closely paralleled HER2 positivity by immunohistochemistry (46.2%) in the cohort. No deletion differed significantly after correction.

Within the surrogate receptor-defined subtypes (5 hormone receptor-positive/HER2-negative, 5 HER2-positive, and 1 triple-negative tumor), none of the 1125 gene-by-stratum comparisons reached FDR < 0.05 under either variant definition (minimum FDR = 0.08; Supplementary Table S5). *PIK3CA* mutation frequencies were similar to those in the corresponding TCGA-BRCA groups (2 of 5 hormone receptor-positive/HER2-negative tumors, 40.0% vs. 38.8%; 1 of 5 HER2-positive tumors, 20.0% vs. 30.7%). *TP53* mutations were more frequent in both major strata, occurring in 4 of 5 HER2-positive tumors (80.0% vs. 36.9%; *p* = 0.07) and 2 of 5 hormone receptor-positive/HER2-negative tumors (40.0% vs. 17.8%; *p* = 0.22), although neither difference was statistically significant.

### 3.4. Whole-Exome Sequencing and aCGH

Whole-exome sequencing was performed on the two pure IMPC tumors with matched normal tissue. Forty-three and 44 coding somatic variants were identified in the two tumors, respectively (Supplementary Table S3). The first harbored 41 nonsynonymous and 2 nonsense substitutions, whereas the second harbored 41 nonsynonymous substitutions, 2 nonsense substitutions, and 1 splice-site variant.

No gene was mutated in both exomes. Genes listed in the Cancer Gene Census were *ERBB2* (p.D582H), *LPP*, *PTPRT,* and *TSC2* in the first tumor and *ARID2* and *ARNT* in the second. Other mutated genes included *MSH3* and *DMBT1*. Array comparative genomic hybridization showed gain of 8q in both tumors, partial in the second, and gain of the 17q region containing *ERBB2* in the first (Supplementary Figure S1).

### 3.5. Whole-Transcriptome Sequencing

Whole-transcriptome sequencing was performed on nine tumors, including two pure IMPCs, at two sequencing sites. Principal-component analysis showed no association between sequencing site and PC1 and only a nominal association with PC2 (Supplementary Figure S2). HER2-positive tumors did not segregate by sequencing site. Unsupervised clustering likewise showed no clear site-specific separation, although some residual site-related structure could not be excluded.

The TCGA-anchored PAM50 procedure assigned three tumors to the HER2-enriched subtype, corresponding exactly to the three HER2-positive tumors by immunohistochemistry. Their centroid correlations ranged from 0.51 to 0.76, with margins of 0.28–0.46, whereas the normal-like call and one Luminal B call (MPC14) had the lowest margins (0.03 and 0.05, respectively). *ERBB2* transcript abundance was higher in HER2-positive than in HER2-negative tumors (median, 16.43 vs. 12.98 on the variance-stabilized scale; Wilcoxon rank-sum *p* = 0.024). The single-sample AIMS classifier agreed with the PAM50 assignment in five of nine tumors. Among the discordant cases, AIMS classified one PAM50 HER2-enriched tumor (MPC6) as Luminal B and one PAM50 basal-like tumor (MPC8) as HER2-enriched; the remaining discordances involved the normal-like and low-confidence luminal calls (Table 2; Supplementary Table S7).

All five tumors with panel *ERBB2* amplification were HER2 positive by immunohistochemistry. Among the seven tumors with both panel and transcriptome data, the two with *ERBB2* amplification were both classified as HER2-enriched, whereas none of the five non-amplified tumors were. The third HER2-enriched tumor had no panel data but showed 17q gain by array comparative genomic hybridization (Table 2). Using TCGA-anchored PAM50, DNA copy number, protein expression, and transcriptional subtype were concordant in these cases, although AIMS classified one amplified/HER2-positive tumor as Luminal B.

Consistent with the copy-number findings, differential expression associated with HER2-enriched status was concentrated in the 17q12–q21 amplified region. After adjustment for sequencing site, 128 genes differed at FDR < 0.05, including 10 of the 11 genes examined within 17q12–q21: *ERBB2* (log2 fold change 3.67), *GRB7* (3.52), *PGAP3* (3.10), *STARD3* (3.03), and *CDK12* (2.23); *RARA* (1.35) was the only gene in this set that did not reach significance. Overexpression of genes within this region was maintained across the alternative sequencing-site analyses, with 9–10 of the 11 genes remaining significant (Supplementary Table S6). Because PAM50 classification itself incorporates HER2-related expression, this differential expression contrast is interpreted as internal cross-platform concordance rather than as an independent transcriptional finding.

No gene was significantly correlated with the proportion of micropapillary morphology after multiple-testing correction (minimum FDR = 0.51). Given the small sample size and heterogeneous tumor cellularity, this analysis had limited power and does not exclude a transcriptional program specific to the micropapillary component.

Fourteen fusion transcripts were detected in six of the nine tumors. No fusion pair or partner gene was recurrent (Supplementary Table S4). One fusion, *SUPT6H*–*CDK12*, was detected in a tumor with *ERBB2* amplification.

## 4. Discussion

We profiled 13 breast carcinomas with an invasive micropapillary component using targeted sequencing, whole-exome sequencing, array comparative genomic hybridization, and transcriptome sequencing. The cohort showed the characteristic clinicopathologic features of IMPC, with approximately three-quarters of tumors being node-positive, a median of eight involved lymph nodes, and lymphovascular invasion in all 11 evaluable tumors [1,2,3]. The recurrent driver alterations largely overlapped those of conventional breast carcinoma. *TP53* was the most frequently altered gene, while *PIK3CA* and *GATA3* were also recurrently mutated; frequencies of *PIK3CA*, *GATA3*, *CDH1*, *MAP3K1*, and *PTEN* were broadly comparable with those in TCGA-BRCA. These findings are consistent with previous studies indicating that IMPC shares most of its recurrent drivers with carcinoma of no special type [5,6,9]. Mutation frequencies nevertheless varied across published series: *TP53* small variants were detected in 7 of 11 tumors and *PIK3CA* mutations in 3 of 11 in the present cohort, compared with 5 of 43 and 2 of 41 pure IMPCs, respectively, in the study by Gruel et al. [23], and one mutation in each gene among eight tumors reported by Natrajan et al. [6]. The *PIK3CA* frequency in our cohort was also similar to that in TCGA-BRCA.

Copy-number alteration was a recurrent feature of this cohort. High-level amplification was concentrated in 17q12–q21, with *ERBB2* amplified in 5 of 11 panel-profiled tumors and *CDK12* and *RARA* in two each. Both pure IMPC tumors analyzed by array comparative genomic hybridization showed 8q gain, and one also showed gain of the 17q region containing *ERBB2*. Gains of 8q and 17q have been reported recurrently in previous array-based and genomic studies of IMPC [5,6,23], and the findings in our cohort are consistent with those reports. Because the targeted panel detects high-level amplification from exon read depth but does not assess broad low-amplitude gains, 8q could be evaluated only in the two array-profiled tumors. *ERBB2* amplification was more frequent in our cohort than in TCGA-BRCA (45.5% vs. 11.5%; *p* = 0.006), but the difference was not significant after multiple-testing correction, and enrichment relative to conventional breast carcinoma is therefore not established. Concordance of *ERBB2* amplification with HER2 protein expression and HER2-enriched classification by TCGA-anchored PAM50 supports the biological relevance of the amplification in these tumors, although one amplified/HER2-positive tumor was classified as Luminal B by AIMS, indicating some classifier dependence.

The fusion transcripts followed the pattern reported by Natrajan and colleagues, who found the fusion transcripts in IMPC to be private events [6]: we detected 14 fusions in six of nine tumors, with no recurrent fusion pair or partner gene. Although the fusion events themselves were distinct, *CDK12* appeared as the 3′ partner in both studies, *LASP1*-*CDK12* in theirs and *SUPT6H*-*CDK12* here, in a tumor with focal 17q12 amplification. Taken together with previous studies, these findings support a heterogeneous molecular landscape characterized by alterations in established breast cancer drivers, recurrent copy-number changes involving 8q and 17q, and predominantly private fusion events. Whether these copy-number alterations are specific to IMPC or are shared with conventional breast carcinomas of similar receptor subtype remains uncertain in the absence of a matched comparison cohort.

Four genes, *NOTCH1*, *BRCA2*, *DOCK2*, and *FLT4*, were more frequently mutated than in TCGA-BRCA after correction across the full panel. However, the reference frequencies were all below 3%, so these comparisons were based on only two to four tumors, and most of the contributing variants were missense substitutions of uncertain significance, several of which were also recorded at low frequency in population databases. When both cohorts were restricted to truncating and hotspot variants, no gene remained significant. These findings therefore do not establish enrichment of these genes in IMPC and should be interpreted cautiously in the absence of matched normal DNA. *TP53* mutation frequency was higher numerically in both major receptor-defined subgroups, but only the overall comparison reached nominal significance, which did not remain significant after multiple-testing correction. No gene expression pattern was significantly associated with micropapillary percentage after correction, although this analysis was limited by the small sample size.

Several limitations should be considered. First, this was a nonconsecutive cohort assembled according to the availability of material for molecular profiling, with tumors accrued over several years and analyzed on partially overlapping platforms. Its receptor-subtype composition may therefore not be representative of IMPC more broadly, and comparisons with TCGA-BRCA should be considered exploratory. Second, most tumors were mixed rather than pure IMPC, and bulk profiling may dilute molecular features confined to the micropapillary component; resolving such features would require microdissection or spatial approaches. Third, matched normal DNA was unavailable for the panel-profiled tumors, and TCGA-BRCA differs from our cohort in sequencing platform, variant-calling pipeline, and ancestry. The comparison was based on a shared target-gene set rather than matched callable territories, and gene-level sensitivity was therefore not directly comparable between the panel and reference exomes. Fourth, the transcriptomic analyses included only nine tumors sequenced at two sites, and a residual site effect cannot be excluded. Finally, clinical outcome data were incomplete, precluding meaningful analysis of recurrence and survival.

## 5. Conclusions

Taken together with previous studies, these findings support a heterogeneous molecular landscape in breast carcinomas with an invasive micropapillary component, characterized by driver alterations shared with conventional breast carcinoma, recurrent copy-number changes involving 8q and 17q, and predominantly private fusion events. *ERBB2* amplification showed cross-platform concordance with HER2 protein expression and HER2-enriched classification by TCGA-anchored PAM50, although intrinsic subtype assignment was not fully concordant across classifiers.

## Figures and Tables

**Figure 1:**
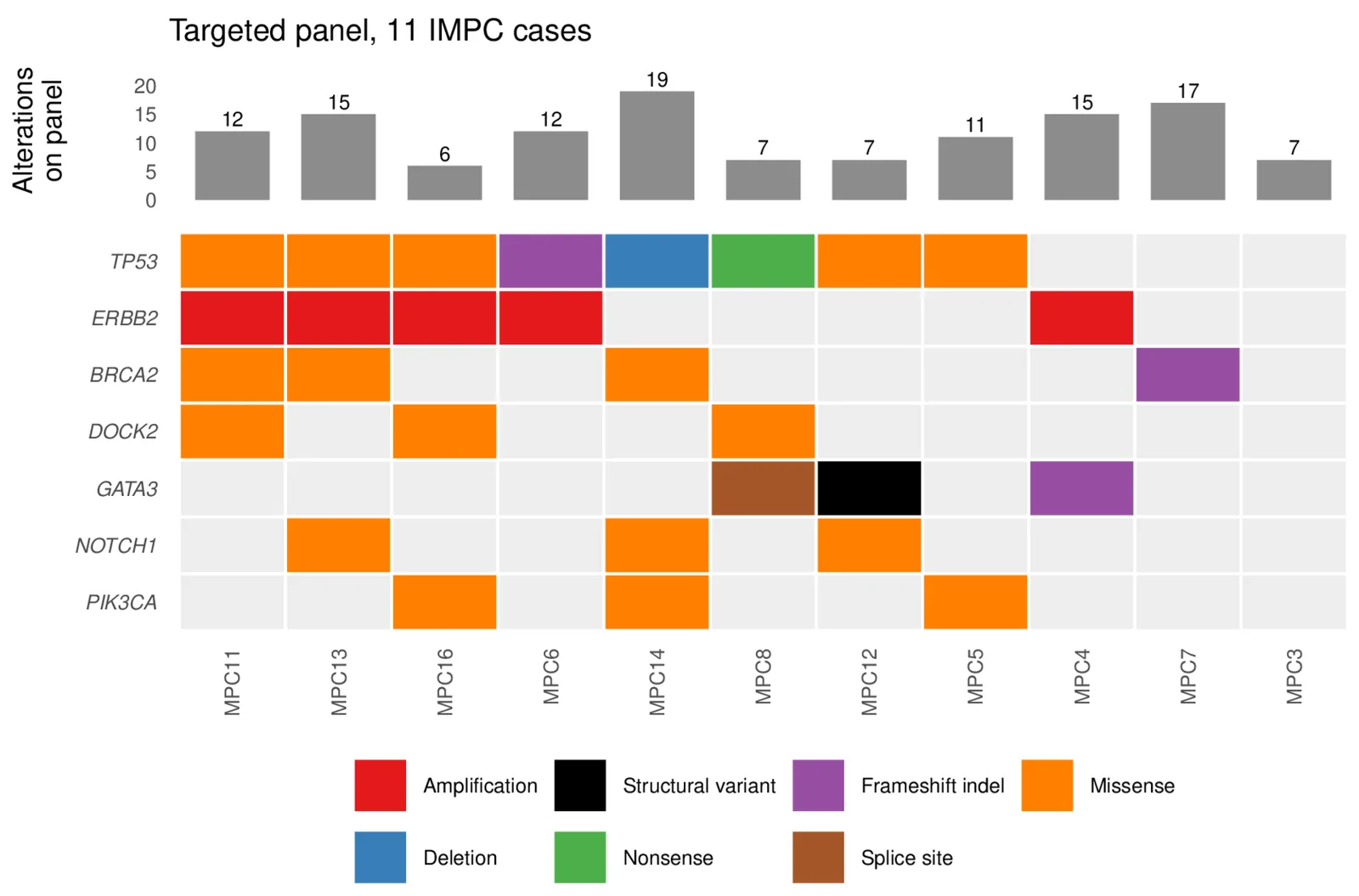
Landscape of genomic alterations in the 11 tumors profiled by targeted deep sequencing. Genes altered in three or more tumors are shown. Color indicates the class of alteration; gray denotes no alteration detected in that gene. The bar above each column gives the total number of panel alterations in that tumor. Every tumor carried at least six alterations, but two carried none in the displayed genes and are therefore gray across the matrix. Where a tumor carried more than one class of alteration in the same gene, copy-number change is shown in preference to a small variant. Copy-number calls were restricted to high-level events as defined in the Methods.

**Figure 2:**
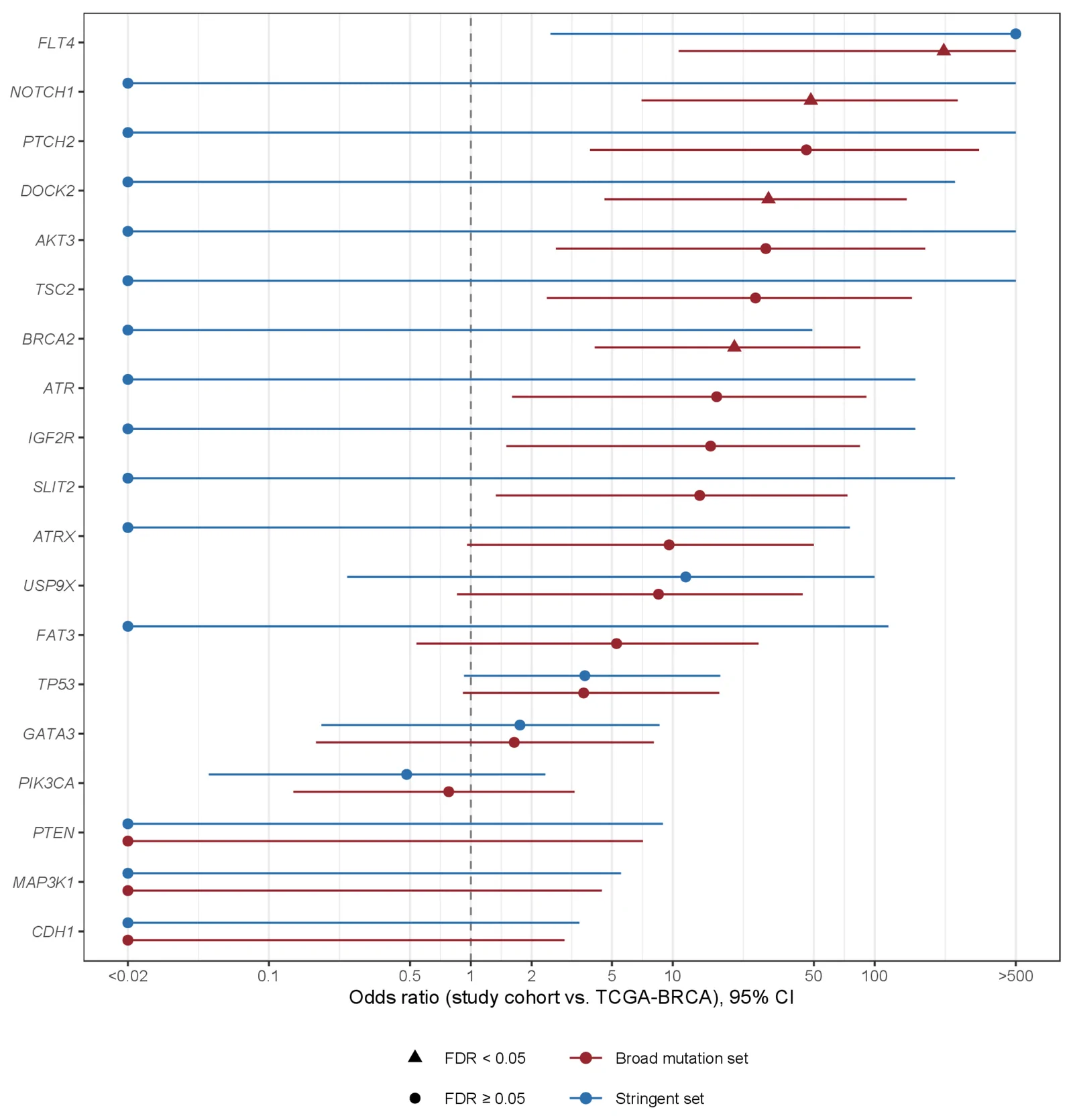
Odds ratios with 95% confidence intervals for protein-altering mutations in the study cohort (*n* = 11) compared with TCGA-BRCA (*n* = 1066). Genes shown are those mutated in at least two tumors in the study cohort or in at least 5% of TCGA-BRCA. Results are shown for the broad mutation set retained after population-frequency filtering and for the stringent mutation set restricted to truncating and hotspot variants, with the same rule applied to TCGA-BRCA. Odds ratios are conditional maximum likelihood estimates from Fisher’s exact test; confidence intervals extending beyond the plotting range are truncated at the axis limits. Triangles indicate comparisons with FDR < 0.05 after Benjamini–Hochberg correction across all 375 panel genes.

**Table 1:** Clinicopathologic characteristics and molecular profiling of the 13 tumors.

Characteristic	Value
**Demographic and pathologic characteristics**	
Age at diagnosis, median (range), years	49 (37–80)
Female sex, *n* (%)	13 (100)
Micropapillary component, median (range), %	40 (10–100)
Pure IMPC, *n* (%)	4 (30.8)
Tumor size, median (range), cm	3.8 (1.2–14.0)ᵃ
Lymphovascular invasion, *n* (%)	11 (100) ᵃ
**Lymph node involvement**	
Node-positive, *n* (%)	10 (76.9)
Positive lymph nodes, median (range), *n*	8 (0–26)
≥4 positive lymph nodes, *n* (%)	8 (61.5)
**Receptor status**	
Hormone receptor positive, *n* (%)	10 (76.9)
HER2 positive, *n* (%)	6 (46.2)
**Molecular profiling**	
Targeted deep sequencing, *n*	11
Whole-exome sequencing and aCGH, *n*	2
Whole-transcriptome sequencing, *n*	9

Bold text indicates category headings. IMPC, invasive micropapillary carcinoma; aCGH, array comparative genomic hybridization. ᵃ tumor size and lymphovascular invasion were each evaluable in 11 of 13 tumors.

**Table 2:** Case-level concordance of *ERBB2* copy-number status, HER2 immunohistochemistry, and intrinsic subtype in tumors with transcriptome data.

Case	Micropapillary Component (%)	Panel *ERBB2*	HER2 IHC	PAM50	AIMS	*ERBB2* Expression
MPC1	100	no panel	+	HER2-enriched	HER2-enriched	16.43
MPC2	100	no panel	−	Normal-like	Luminal A	12.97
MPC3	40	not amplified	−	Luminal A	Luminal A	13.11
MPC4	10	amplified	+	HER2-enriched	HER2-enriched	17.40
MPC5	70	not amplified	−	Luminal B	Luminal B	12.78
MPC6	10	amplified	+	HER2-enriched	Luminal B	16.26
MPC7	30	not amplified	−	Luminal B	Luminal B	13.85
MPC8	20	not amplified	−	Basal-like	HER2-enriched	12.99
MPC14	10	not amplified	−	Luminal B	Luminal A	12.69

PAM50 denotes the TCGA-anchored PAM50 assignment. *ERBB2* expression is shown on the variance-stabilized scale. IHC, immunohistochemistry.

## Data Availability

The processed results supporting the findings of this study are provided within the article and its Supplementary Materials. The analysis code is available from the corresponding authors upon reasonable request.

## Supplementary Materials

The following supporting information can be downloaded at https://www.mdpi.com/article/10.3390/genes17101190/s1, Figure S1: Array comparative genomic hybridization profiles of the two pure IMPC tumors. Each panel shows the log2 tumor-to-reference ratio of array probes along the chromosome, with the chromosome ideogram shown above. Probes are colored according to the copy-number status assigned by GLAD: yellow, balanced; red, gain; blue, high-level gain; and green, loss. Crossed symbols indicate probes flagged as outliers by GLAD. The black step line represents the segment mean, and vertical dashed lines indicate segment boundaries. (A) Chromosome 17 in MPC1, showing gain of the 17q region containing *ERBB2*. (B) Chromosome 8 in MPC1, showing gain of 8q. (C) Chromosome 8 in MPC2, showing partial gain of 8q; Figure S2: Technical structure of the nine transcriptomes. Principal-component analysis of the 2000 most variable genes using variance-stabilized expression values. Tumors are annotated by sequencing site (shape), TCGA-anchored PAM50 subtype (color), and HER2 immunohistochemistry status (point size). (A) PC1 versus PC2, explaining 23.8% and 17.4% of the variance, respectively. (B) PC1 versus PC3, with PC3 explaining 14.2% of the variance. (C) PC1 versus PC2 after removal of the sequencing-site effect. (D) Unsupervised hierarchical clustering using Ward’s method and Euclidean distance, with each tumor labeled by sequencing site and PAM50 subtype; Table S1: Clinicopathologic characteristics and platform coverage of the 13 tumors; Table S2: Per-sample sequencing quality metrics by assay; Table S3: Case-level small variants from the targeted panel and from whole-exome sequencing; Table S4: Case-level structural variants and fusion transcripts detected by targeted panel and RNA sequencing; Table S5: Gene-level comparison of genomic alterations between IMPC and TCGA-BRCA; Table S6: Differential expression of 17q12–q21 genes in HER2-enriched tumors; Table S7: Intrinsic subtype assignments by TCGA-anchored PAM50 and AIMS; Data S1: Gene-level RNA-seq read counts for nine tumors and two matched normal samples.

## References

[1] Verras G.-I., Tchabashvili L., Mulita F., Grypari I.M., Sourouni S., Panagodimou E., Argentou M.-I. (2022). Micropapillary Breast Carcinoma: From Molecular Pathogenesis to Prognosis. Breast Cancer.

[2] Nassar H., Wallis T., Andea A., Dey J., Adsay V., Visscher D. (2001). Clinicopathologic Analysis of Invasive Micropapillary Differentiation in Breast Carcinoma. Mod. Pathol..

[3] Acs G., Esposito N.N., Rakosy Z., Laronga C., Zhang P.J. (2010). Invasive Ductal Carcinomas of the Breast Showing Partial Reversed Cell Polarity are Associated With Lymphatic Tumor Spread and may Represent Part of a Spectrum of Invasive Micropapillary Carcinoma. Am. J. Surg. Pathol..

[4] Perron M., Wen H.Y., Hanna M.G., Brogi E., Ross D.S. (2021). HER2 Immunohistochemistry in Invasive Micropapillary Breast Carcinoma: Complete Assessment of an Incomplete Pattern. Arch. Pathol. Lab. Med..

[5] Marchiò C., Iravani M., Natrajan R., Lambros M., Savage K., Tamber N., Fenwick K., Mackay A., Senetta R., Di Palma S. (2008). Genomic and immunophenotypical characterization of pure micropapillary carcinomas of the breast. J. Pathol..

[6] Natrajan R., Wilkerson P.M., Marchiò C., Piscuoglio S., Ng C.K., Wai P., Lambros M.B., Samartzis E.P., Dedes K.J., Frankum J. (2014). Characterization of the genomic features and expressed fusion genes in micropapillary carcinomas of the breast. J. Pathol..

[7] Lv J., Shi Q., Han Y., Li W., Liu H., Zhang J., Niu C., Gao G., Fu Y., Zhi R. (2021). Spatial transcriptomics reveals gene expression characteristics in invasive micropapillary carcinoma of the breast. Cell Death Dis..

[8] Shi Q., Shao K., Jia H., Cao B., Li W., Dong S., Liu J., Wu K., Liu M., Liu F. (2022). Genomic alterations and evolution of cell clusters in metastatic invasive micropapillary carcinoma of the breast. Nat. Commun..

[9] Dieci M.V., Smutná V., Scott V., Yin G., Xu R., Vielh P., Mathieu M.-C., Vicier C., Laporte M., Drusch F. (2016). Whole exome sequencing of rare aggressive breast cancer histologies. Breast Cancer Res. Treat..

[10] Wolff A.C., Hammond M.E.H., Allison K.H., Harvey B.E., Mangu P.B., Bartlett J.M., Bilous M., Ellis I.O., Fitzgibbons P., Hanna W. (2018). Human Epidermal Growth Factor Receptor 2 Testing in Breast Cancer: American Society of Clinical Oncology/College of American Pathologists Clinical Practice Guideline Focused Update. Arch. Pathol. Lab. Med..

[11] Hammond M.E.H., Hayes D.F., Dowsett M., Allred D.C., Hagerty K.L., Badve S., Fitzgibbons P.L., Francis G., Goldstein N.S., Hayes M. (2010). American Society of Clinical Oncology/College of American Pathologists guideline recommendations for immunohistochemical testing of estrogen and progesterone receptors in breast cancer. Arch. Pathol. Lab. Med..

[12] Lee H., Cho Y.A., Kim D.G., Cho E.Y. (2024). Next-Generation Sequencing in Breast Cancer Patients: Real-World Data for Precision Medicine. Cancer Res. Treat..

[13] Kidd J.M., Newman T.L., Tuzun E., Kaul R., Eichler E.E. (2007). Population Stratification of a Common APOBEC Gene Deletion Polymorphism. PLoS Genet..

[14] Chang M.T., Bhattarai T.S., Schram A.M., Bielski C.M., Donoghue M.T., Jonsson P., Chakravarty D., Phillips S., Kandoth C., Penson A. (2018). Accelerating discovery of functional mutant alleles in cancer. Cancer Discov..

[15] Love M.I., Huber W., Anders S. (2014). Moderated estimation of fold change and dispersion for RNA-seq data with DESeq2. Genome Biol..

[16] Parker J.S., Mullins M., Cheang M.C., Leung S., Voduc D., Vickery T., Davies S., Fauron C., He X., Hu Z. (2009). Supervised risk predictor of breast cancer based on intrinsic subtypes. J. Clin. Oncol..

[17] Hoadley K.A., Yau C., Hinoue T., Wolf D.M., Lazar A.J., Drill E., Shen R., Taylor A.M., Cherniack A.D., Thorsson V. (2018). Cell-of-Origin Patterns Dominate the Molecular Classification of 10,000 Tumors from 33 Types of Cancer. Cell.

[18] Paquet E.R., Hallett M.T. (2015). Absolute assignment of breast cancer intrinsic molecular subtype. J. Natl. Cancer Inst..

[19] Gendoo D.M.A., Ratanasirigulchai N., Schröder M.S., Paré L., Parker J.S., Prat A., Haibe-Kains B. (2016). Genefu: An R/Bioconductor package for computation of gene expression-based signatures in breast cancer. Bioinformatics.

[20] Cerami E., Gao J., Dogrusoz U., Gross B.E., Sumer S.O., Aksoy B.A., Jacobsen A., Byrne C.J., Heuer M.L., Larsson E. (2012). The cBio cancer genomics portal: An open platform for exploring multidimensional cancer genomics data. Cancer Discov..

[21] Gao J., Aksoy B.A., Dogrusoz U., Dresdner G., Gross B.E., Sumer S.O., Sun Y., Jacobsen A., Sinha R., Larsson E. (2013). Integrative analysis of complex cancer genomics and clinical profiles using the cBioPortal. Sci. Signal..

[22] Cancer Genome Atlas Network (2012). Comprehensive molecular portraits of human breast tumours. Nature.

[23] Gruel N., Benhamo V., Bhalshankar J., Popova T., Fréneaux P., Arnould L., Mariani O., Stern M.-H., Raynal V., Sastre-Garau X. (2014). Polarity gene alterations in pure invasive micropapillary carcinomas of the breast. Breast Cancer Res..

