# Supplementary material for: Multiplatform Genomic and Transcriptomic Profiling of Breast Carcinomas with an Invasive Micropapillary Component
Source: Genes (Basel). 2026 Sep 25;17(10):1190. doi: 10.3390/genes17101190 (PMC13704641; doi:10.3390/genes17101190)
Supplement: Supplementary file 1 [file genes-17-01190-s001.zip › Figure S1_aCGH.pdf]

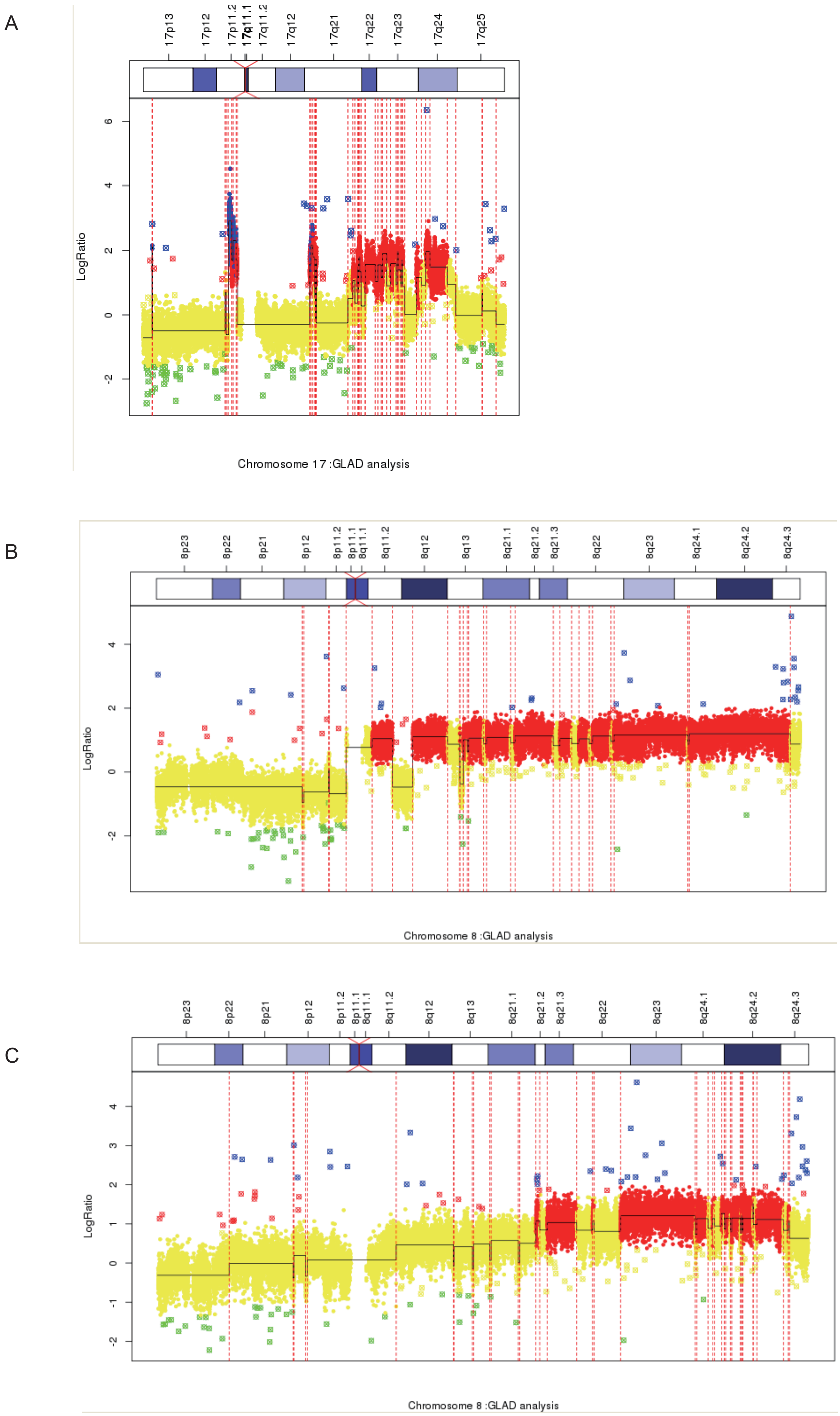

A
17p13
17p12
17p11.2
17q11.1
17q11.2
17q12
17q21
17q22
17q23
17q24
17q25
LogRatio
Chromosome 17 :GLAD analysis
B
8p23
8p22
8p21
8p12
8p11.2
8p11.1
8q11.1
8q11.2
8q12
8q13
8q21.1
8q21.2
8q21.3
8q22
8q23
8q24.1
8q24.2
8q24.3
LogRatio
Chromosome 8 :GLAD analysis
C
8p23
8p22
8p21
8p12
8p11.2
8p11.1
8q11.1
8q11.2
8q12
8q13
8q21.1
8q21.2
8q21.3
8q22
8q23
8q24.1
8q24.2
8q24.3
LogRatio
Chromosome 8 :GLAD analysis
