# Supplementary material for: Multiplatform Genomic and Transcriptomic Profiling of Breast Carcinomas with an Invasive Micropapillary Component
Source: Genes (Basel). 2026 Sep 25;17(10):1190. doi: 10.3390/genes17101190 (PMC13704641; doi:10.3390/genes17101190)
Supplement: Supplementary file 1 [file genes-17-01190-s001.zip › Figure S2_PCA_batch.pdf]

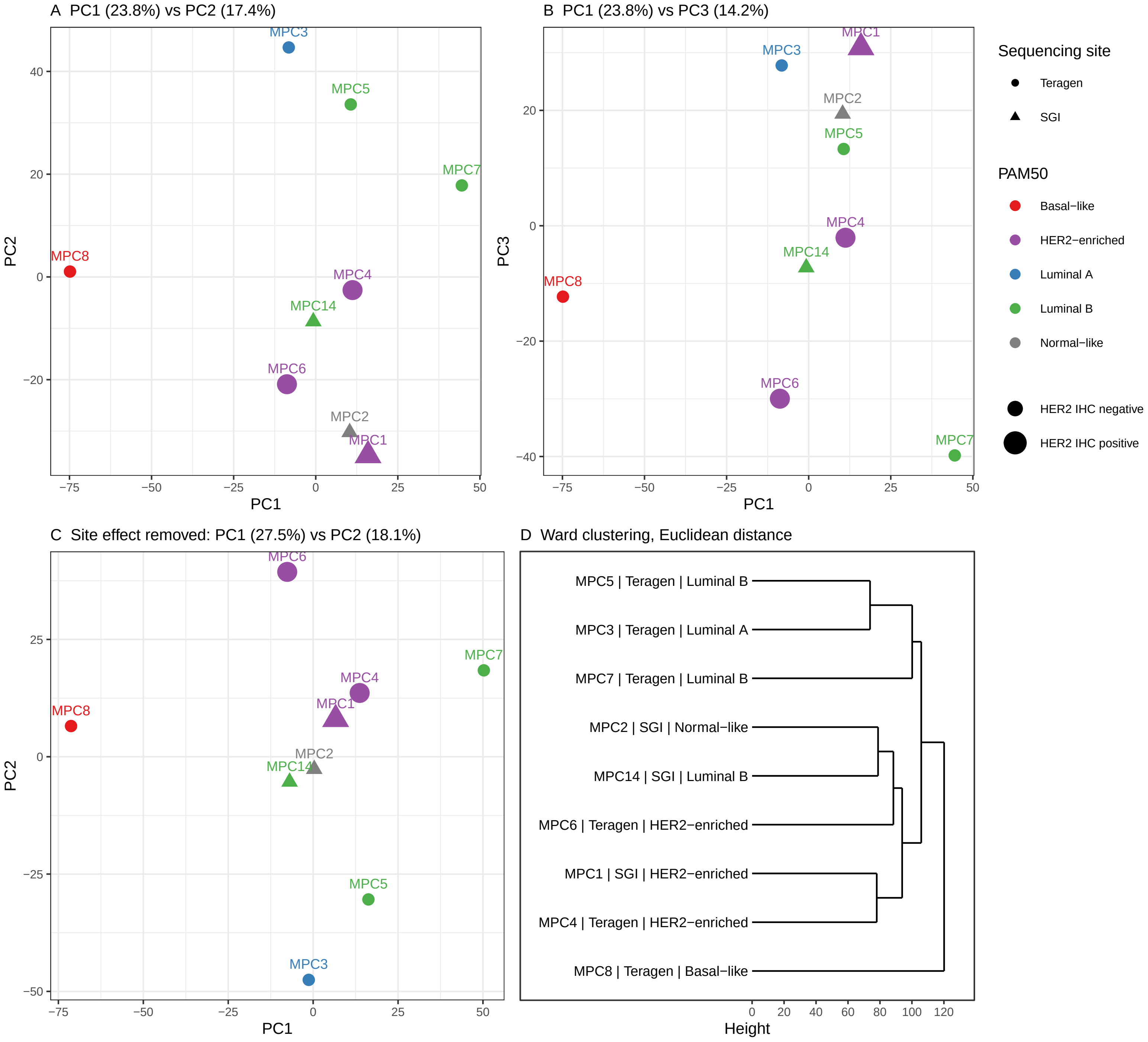

A PC1 (23.8%) vs PC2 (17.4%)
B PC1 (23.8%) vs PC3 (14.2%)
C Site effect removed: PC1 (27.5%) vs PC2 (18.1%)
D Ward clustering, Euclidean distance
PC1
PC2
PC3
Height
MPC1
MPC2
MPC3
MPC4
MPC5
MPC6
MPC7
MPC8
MPC14
Sequencing site
Teragen
SGI
PAM50
Basal−like
HER2−enriched
Luminal A
Luminal B
Normal−like
HER2 IHC negative
HER2 IHC positive
MPC5 | Teragen | Luminal B
MPC3 | Teragen | Luminal A
MPC7 | Teragen | Luminal B
MPC2 | SGI | Normal−like
MPC14 | SGI | Luminal B
MPC6 | Teragen | HER2−enriched
MPC1 | SGI | HER2−enriched
MPC4 | Teragen | HER2−enriched
MPC8 | Teragen | Basal−like
